# Seizures in childhood cerebral adrenoleukodystrophy: New insights and remaining knowledge gaps

**DOI:** 10.1111/dmcn.16352

**Published:** 2025-05-15

**Authors:** Samuel Groeschel, Hendrik Rosewich

**Affiliations:** ^1^ Department of Child Neurology, General Pediatrics, Endocrinology, Diabetology, Social Pediatrics University Hospital and Faculty of Medicine, Eberhard Karls University Tübingen Tübingen Germany

## Abstract

This commentary is on the original article by Page et al. on pages 1494–1500 of this issue.

Seizures in X‐linked adrenoleukodystrophy (X‐ALD) often reflect progressive cerebral involvement, typically emerging as the disease advances and demyelination intensifies, particularly in the childhood cerebral form of the disorder (CCALD). However, important knowledge gaps remain, including the factors predicting seizure onset, frequency, and severity in different phenotypic presentations of X‐ALD. The current study by Page et al.^1^ sheds light on the clinical presentation and the seminal relationships between seizures and the clinical course of CCALD, an inflammatory demyelinating disease resulting from mutations in the X‐linked *ABCD1* gene. By analyzing a cohort of 86 male children diagnosed with CCALD, including 25 who experienced seizures, the authors established a significant correlation between seizure occurrence and increased disease severity, as indicated by higher magnetic resonance imaging (MRI) lesion burden (Loes score) and greater functional impairment. Seizures were not observed in patients with Loes scores below 6 and were increasingly prevalent in individuals with more extensive disease burden. Among 53 adult males with X‐ALD but no detectable MRI abnormalities, only three had a history of seizures – a prevalence marginally exceeding that of the general population. These findings suggest that cerebral involvement is the primary factor contributing to epileptogenicity in X‐ALD.

Despite these insights, the study also underscores substantial knowledge gaps. Notably, 10 male children were diagnosed with ALD following seizure onset, and one patient experienced a seizure 7 years prior to the detection of cerebral lesions on MRI. While most seizures appear to emerge alongside disease progression, these outliers raise intriguing questions about early, subclinical changes or other factors that may trigger seizures. The pathophysiology of seizures in ALD is likely multifactorial. Although white matter inflammation is a well‐established contributor, secondary cortical gray matter pathology, metabolic disturbances associated with peroxisomal dysfunction, and adrenal insufficiency^2^ may also be involved. Further investigation is warranted to elucidate these potential mechanisms.

The study also highlights the absence of disease‐specific guidelines for seizure management in X‐ALD. Currently, clinicians rely on general epilepsy treatment principles,^3^ despite the distinct neuropathological characteristics of X‐ALD. Furthermore, the broader impact of seizures – beyond their association with lesion severity – on family burden and overall quality of life remains insufficiently characterized. Given the already debilitating progression of the disease, the unpredictability of seizures may substantially exacerbate caregiver stress.

These findings have important clinical implications. First, seizures may be more prevalent in CCALD than previously recognized and can manifest early in the disease course. Second, in young males with a known diagnosis of X‐ALD, seizure onset should be considered a critical indicator of disease progression and should prompt an immediate reassessment of treatment eligibility. Given that hematopoietic stem cell transplantation and gene therapy are only indicated at early stages (typically Loes scores ≤9),^4^ the appearance of seizures may indicate a narrowing treatment window.

Finally, Page et al. present valuable data derived from a relatively large cohort within a rare disease population, offering practical insights while simultaneously highlighting the existing gaps in our understanding of seizure mechanisms in X‐ALD. Further prospective and mechanistic studies are needed – not only to optimize seizure management but also to elucidate their role in disease progression. In CCALD, seizures are not merely a symptom but may serve as a critical indicator of the underlying pathophysiological processes driving disease progression.

## Data Availability

Not required.
